# The Effect of Xylooligosaccharide, Xylan, and Whole Wheat Bran on the Human Gut Bacteria

**DOI:** 10.3389/fmicb.2020.568457

**Published:** 2020-12-03

**Authors:** Miao Chen, Shujun Liu, Khandaker Md. Sharif Uddin Imam, Lichao Sun, Yulu Wang, Tianyi Gu, Boting Wen, Fengjiao Xin

**Affiliations:** Laboratory of Biomanufacturing and Food Engineering, Institute of Food Science and Technology, Chinese Academy of Agricultural Sciences, Beijing, China

**Keywords:** ferulic acid, human gut microbiota, wheat bran, *in vitro* fermentation, Illumina MiSeq sequencing

## Abstract

Wheat bran is a cereal rich in dietary fibers that have high levels of ferulic acid, which has prebiotic effects on the intestinal microbiota and the host. Herein we explored the effect of xylooligosaccharide, xylan, and whole wheat bran on the human gut bacteria and screened for potential ferulic acid esterase genes. Using *in vitro* fermentation, we analyzed the air pressure, pH-value, and short-chain fatty acid levels. We also performed 16S rRNA gene and metagenomic sequencing. A Venn diagram analysis revealed that 80% of the core operational taxonomic units (OTUs) were shared among the samples, and most of the xylooligosaccharide treatment core OTUs (319/333 OTUs) were shared with the other two treatments’ core OTUs. A significant difference analysis revealed that the relative abundance of *Dorea*, *Bilophila*, and *Sulfurovum* in wheat bran treatment was higher than that in xylan and xylooligosaccharide treatments. The clusters of orthologous groups of proteins functional composition of all samples was similar to the microbiota composition of the control. Using metagenomic sequencing, we revealed seven genes containing the conserved residues, Gly-X-Ser-X-Gly, and the catalytic triad, Ser-His-Asp, which are thus potential ferulic acid esterase genes. All the results indicate that xylan and/or xylooligosaccharide, the main dietary fibers in wheat bran, plays a major role in *in vitro* fermentation by the human gut microbiota.

## Introduction

The human intestinal microbiota has up to 39 trillion microorganisms. The interactions of these microbes with each other and with the host regulate biological processes that are vital to human health (Makki et al., 2018; Tierney et al., 2019). Diet is not only essential for maintaining human growth and health, but it is also the main energy source for the growth of the intestinal microbiota. In particular, dietary fiber can be selectively digested by the human gut microbiota, which has a positive effect on promoting the production of beneficial metabolites and maintaining the diversity of gut microbiota (Fang et al., 2019). The incidence and the prevalence of obesity, diabetes, and cardiovascular and cerebrovascular diseases are rapidly increasing (Woting and Blaut, 2016; Oh et al., 2019). An important reason for this is the refined processing of cereals, the increase in the glycemic index of foods, and the severe loss of dietary fiber. Fortunately, wheat bran (WB) is rich in dietary fiber, which accounts for 47.2 ± 1.4% of WB on a dry basis (Yang et al., 2014), and 74% of the dietary fiber comprises xylan (X) and xylooligosaccharide (XO) (Manisseri and Gudipati, 2010).

An increasing number of studies have focused on the effect of X, XO, and WB on the intestinal microbiota. De Paepe et al. (2018) have used the Simulator of the Human Intestinal Microbial Ecosystem to study the effects of insoluble WB particles on the structure and the function of intestinal microbiota. They found that WB in the intestine forms a new niche for microbiota enrichment. This has a positive effect on maintaining microbial diversity and promoting the production of beneficial metabolites (De Paepe et al., 2018). Furthermore, WB reduced the levels of the *Clostridium* and *Turicibacter* genera and strongly augmented the abundance of *Bifidobacterium* and *Butyricicoccus* (Suriano et al., 2017). Additionally, Yang et al. (2014) have studied the *in vitro* fermentation of polysaccharides (feruloylated oligo- and polysaccharides) from WB, though they only assayed *Bifidobacterium*. Moreover, as the main WB dietary fiber, XO can increase the percentage of *Bacteroides* and the yield of butyric acid and acetic acid (Miguez et al., 2018) and relieve autoimmune symptoms in NOD mice (Hansen et al., 2019). Moreover, a structural analysis of the intestinal microbiota of mice administered a western diet showed that arabinoxylan oligosaccharides in WB were the most effective for weight loss (Suriano et al., 2017). Suriano et al. (2017) have speculated that arabinoxylan oligosaccharides in WB are the key nutrients for prebiotics. However, no study has focused on the effect of X on the intestinal microbiota. Herein we studied whether XO and/or X in WB plays a vital role in the gut microbiota.

Polyphenols are beneficial for preventing and treating inflammatory bowel disease and regulating intestinal microbiota (Tang et al., 2020). Ferulic acid (FA), which is a type of hydroxyphenyl acrylic acid, exists on the side chain of X, whose content is 63.0–445 mg/100 g WB, which is second only to its content in corncob (Fava et al., 2013; Kumar and Pruthi, 2014). The total conversion rate of FA is 93.5% (Ferri et al., 2019). However, under optimized conditions at the bioreactor scale, the maximum FA yield obtained is 0.82–1.05 g FA/kg bran. Compared with the total FA content in WB, this still represents a minor advancement in the FA extraction rate (Ferri et al., 2019). Therefore, WB can be used as a raw material for the preparation of FA, which is important for mining FA esterase (FAE) with high enzyme activity as it improves the extraction rate of FA. The catalytic triad and the conserved residues, Gly-X-Ser-X-Gly, are two typical characteristics of FAEs, which belong to carbohydrate esterase family 1 (CE1). The human gut microbiota is rich in enzymes and microorganisms and can be used for extracting resources (Yu et al., 2020), thus providing the possibility to explore novel FAEs.

This study provides a basis for screening novel FAEs and lays a theoretical foundation for improving FA extraction yield from WB. Furthermore, understanding whether X and/or XO in WB plays a vital role in the gut microbiota will provide a theoretical basis for its nutritional value.

## Materials and Methods

**FIGURE 1:**
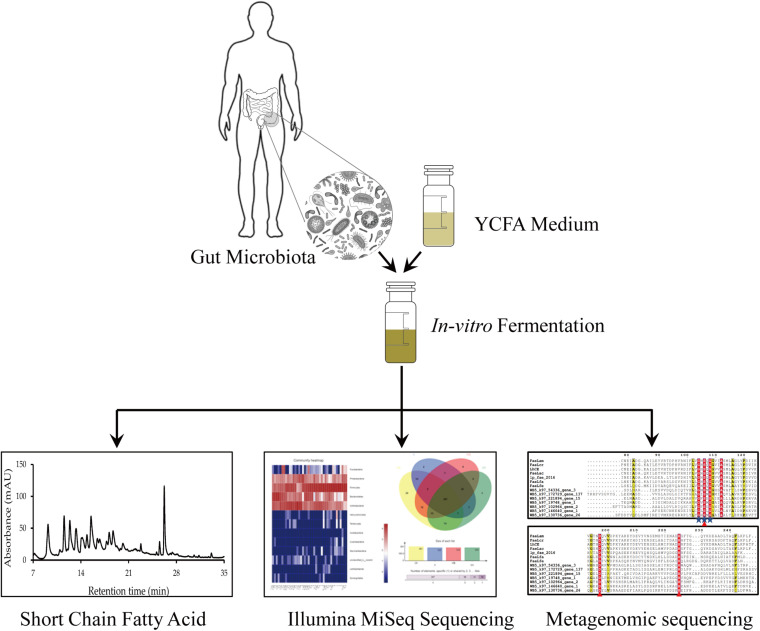
The *in vitro* fermentation strategy followed by the main methods of analysis.

In this study, X, XO, and WB fermentation was assessed simultaneously. Our results elucidated the similarities and the differences in the effects of the three substrates on the intestinal microbiota. Furthermore, a high-throughput sequencing method was used to sequence human intestinal microbiota to compare the microbial community structures after *in vitro* fermentation with WB, X, and XO as substrates. We attempted to clarify the effects of WB, X, and XO on the human gut microbiota. Figure 1 depicts the proposed research strategy. The production of FA and short-chain fatty acids (SCFAs), as well as the gas yield and the pH-value, was measured. Additionally, metagenomic sequencing was performed to mine potential FAE genes. Species and functional annotation of genes was assessed according to the CAZy database. Potential FAEs will be expressed and purified, and their characteristics will be determined in future experiments.

### Substrate Characteristics

WB was provided by a farm in Hebei Province, China. X (85% purity grade) was purchased from Aladdin Reagent Co., China. XO (99% purity grade) was obtained from Heagreen Company, China. X and XO were extracted from corn. WB, X, and XO were grounded and passed through a 0.22-mm sieve without further treatment. The content of cellulose and hemicellulose in WB was measured by high-performance liquid chromatography (HPLC) according to the method of the National Renewable Energy Laboratory. All treatments were performed in triplicate. The cellulose content in WB was 17.88 ± 0.49%, and the hemicellulose content was 10.42 ± 0.2% (Supplementary Table S1). According to the data, the polysaccharide content in WB was up to 28.3%.

### Fecal Inoculum Collection and Preparation

Human fecal samples were obtained from 10 healthy donors (five females and five males) who were not treated with antibiotics for more than 2 months before the trial. All the participants were recruited by the following inclusion criteria: age between 22 and 36 years, Chinese citizens, non-smoking, and healthy. The exclusion criteria included clinically significant deviations from normal based on the investigators’ judgment, history or suspicion of diabetes, liver disease, kidney disease, having a food allergy, or taking dietary fiber supplements or lipid-lowering drugs. The inoculum was prepared by suspending the fresh fecal sample in 0.1 M phosphate buffer (pH 6.5, 0.2 M NaH_2_PO_4_, and 0.1 M Na_2_HPO_4_) supplemented with 1.0% L-ascorbic acid. The concentration of the fecal suspension was 10% (w/v).

This study was carried out following the recommendations of the provisions in Article 11 of the “Ethics Review Methods for Human-Related Biomedical Research (Draft for Soliciting Opinions)” (National Health and Family Planning Commission of China). The protocol was approved by the Human Research Ethics Committee of the Institute of Food Science and Technology, the Chinese Academy of Agricultural Sciences. All the subjects gave written informed consent based on the Declaration of Helsinki.

### *In vitro* Fermentation

Each substrate was weighed into an autoclaved vial containing 5 ml of culture medium, filled with N_2_. Fresh excrement from 10 donors who had not taken dietary fiber supplements was used to make a 10% (w/v) fecal slurry, and each substrate was fermented in 10 duplicates. Using a 1-ml syringe, 0.5 ml of fecal slurry was inoculated into the culture medium and incubated at 37°C for 24 h for fermentation. Samples were then harvested, and the pH, gas, SCFAs, and intestinal microbiota were analyzed (Chen et al., 2020).

Every fermented fecal slurry vial had 5 ml of YCFA medium (Schwab et al., 2017) added with 1% insoluble substrate (WB) or 1% soluble prebiotic substrates (X and XO). The control group had no prebiotics (CK group). These media were autoclaved at 121°C for 20 min. Using a mixture of N_2_ and CO_2_ (80:20; 0.02–0.04 MPa), we obtained anaerobic conditions. The gas mixture was filtered through a 0.2-μm polytetrafluoroethylene membrane for sterilization. The fecal suspension (500 μl) was inoculated into the corresponding vial.

After 24 h of cultivation, air pressure in the WB, X, XO, and CK groups was measured by a BMP-Test System pressure gauge (WAL Mess- und Regelsysteme GmbH, Oldenburg, Germany). The pH-value of the supernatant was determined by a compact pH meter (Model B-212, Horiba, Japan). The samples were stored at –20°C.

### SCFA Analysis

The harvested samples (500 μl) were mixed with 100 μl crotonic acid, incubated at −20°C for 12 h, and centrifuged (16,060 *g* for 3 min), and 100 μl was injected onto a GC-9270 (Zhejing Fuli Analytical Instrument Co., Ltd.) attached to a HP-FFAP column (30 m × 0.25 mm × 0.25 μm; Agilent Technologies Inc., Santa Clara, CA, United States). Using external calibration curves, acetic acid, propionic acid, butyric acid, isobutyric acid, valeric acid, and isovaleric acid in the samples were quantified (for details, please refer to Chen et al., 2020).

### 16S Sequencing

Genomic DNA from WB, X, XO, and CK-treated samples was extracted for 16S sequencing. The methods used were according to the corresponding manufacturer’s instructions (for details, please refer to our previous study; Chen et al., 2020). The raw reads used in this study have been deposited into the NCBI Sequence Read Archive database (accession no. PRJNA577201).

### FA Content Analysis

HPLC was used to determine the FA content in the supernatant of the fermentation system. The supernatant of the fermentation broth and absolute ethanol were mixed in a 1:1 ratio and passed through a 0.22-μm filter membrane, and 0.5 ml was transferred into a brown injection bottle. The FA content was determined by HPLC (Wang et al., 2020; Yao et al., 2020).

### Metagenomic Sequencing

Sample 5_WB was selected from the 20 samples for further metagenomic analysis, which was conducted at Majorbio Bio-Pharm Technology Co., Ltd. (Shanghai, China) (Zheng et al., 2020). The concentration and the purity of the extracted DNA were determined using the TBS-380 and NanoDrop 2000 instruments, respectively. Paired-end sequencing was performed using Illumina NovaSeq (Illumina Inc., San Diego, CA, United States). Adapter sequences were stripped from the 3′ and 5′ ends of the paired-end Illumina reads using SeqPrep^1^. Low-quality reads (length < 50 bp or with a quality value < 20 or having N bases) were removed using Sickle^2^. The reads were aligned to the human genome using BWA^3^, and any hits associated with the reads and their mated reads were removed. Metagenomics data were assembled using MEGAHIT (Li et al., 2015), which uses succinct de Bruijn graphs. Contigs with lengths over 300 bp were selected as the final assembling results, which were then used for further gene prediction and annotation. Open reading frames (ORFs) from each assembled contig were predicted using MetaGene (Noguchi et al., 2006). The predicted ORFs with lengths over 100 bp were retrieved and translated into amino acid sequences using the NCBI translation table.

All predicted genes with 95% sequence identity (90% coverage) were clustered using CD-HIT (Fu et al., 2012), and the longest sequences, from each cluster, were selected as representative sequences to construct a non-redundant gene catalog. Reads after quality control were mapped to representative sequences with 95% identity using SOAPaligner (Li et al., 2008)^4^, and gene abundance in each sample was evaluated. Carbohydrate-active enzymes were annotated using hmmscan^5^ against CAZy database, version 5.0^6^, with an e-value cutoff of 1e^–5^.

### Statistical Analysis

Data processing and statistical analyses of physicochemical properties were performed using GraphPad Prism 7.0.4 software, and all data are expressed as mean ± SD. Assessments between groups were analyzed using one-way ANOVA, followed by Kruskal–Wallis test. 16S sequencing data analysis was performed in the platform of Major Bio Information Cloud Platform. All sequences were clustered into operational taxonomic units (OTUs) based on 97% identity threshold by the SILVA database (Quast et al., 2013). A result was considered statistically significant when the *P*-value was less than 0.05.

## Results

### Physicochemical Property Analysis

The barometric pressure, pH, and SCFAs in *in vitro* anaerobic fermentation, which are associated with microbiota activity, were assayed after 24 h. The barometric pressure hardly changed from 24 to 48 h. Therefore, we determined the physicochemical property changes only at 24 h. This duration represents the time that food stays in the human body. The barometric pressure, pH, and SCFA yield data for each treatment are presented as the average of 10 samples per treatment.

### Air Pressure and pH Analysis

The air pressure in the WB, X, XO, and CK groups is shown in Supplementary Figure S1. Compared with the CK group (8 kPa), the barometric pressure of the three substrates (WB, X, and XO) increased and reached a similar level (24, 25, and 22 kPa, respectively).

**FIGURE 2:**
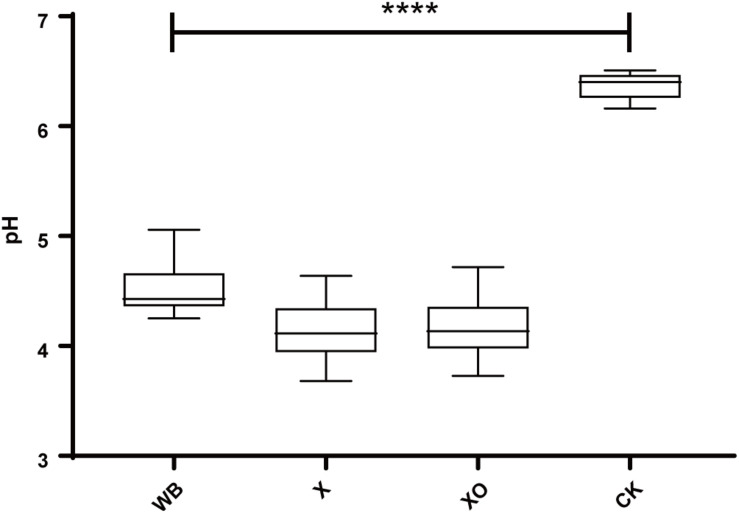
The pH of the *in vitro* fermentation after 24 h. The significance of the differences in pH was calculated by Kruskal–Wallis test; ****P < 0.0001 compared with the control.

Figure 2 shows the pH results. Compared with the control, the pH-value of the three treatments was significantly reduced after 24 h of *in vitro* anaerobic fermentation. The pH of the WB treatment was slightly higher than that of the X and XO treatments (4.5, 4.1, and 4.2, respectively).

### SCFA Analysis

Measuring the SCFA yield indirectly infers the effects of WB, X, and XO on the intestinal microbiota and the host. SCFA production after *in vitro* anaerobic fermentation is shown in Supplementary Figure S2. The SCFA production was based on the weight of dry matter. Notably, there was a small amount of SCFAs in the CK group. Acetic acid was the main SCFA in *in vitro* anaerobic fermentation (Supplementary Figure S2A); the acetic acid yield in the XO group was higher than that in the WB and X groups, which was 3,720, 2,245, and 3,086 μmol/g, respectively. Propionic acid production in the WB, X, and XO groups was 414, 386, and 560 μmol/g, respectively (Supplementary Figure S2B). Butyric acid production was 296, 241, and 506 μmol/g, respectively (Supplementary Figure S2C). Additionally, when including isobutyric acid, valeric acid, and isovaleric acid, the total average of SCFAs in the three substrate groups was 3,904, 3,216, and 5,026 μmol/g, respectively (Supplementary Figure S2D), namely, the SCFAs were increased after the addition of the three substrates. The XO treatment induced more SCFA production than the WB treatment did, and both treatments induced a higher SCFA production than X treatment did.

### 16S rRNA Gene Amplicon Analysis

**FIGURE 3:**
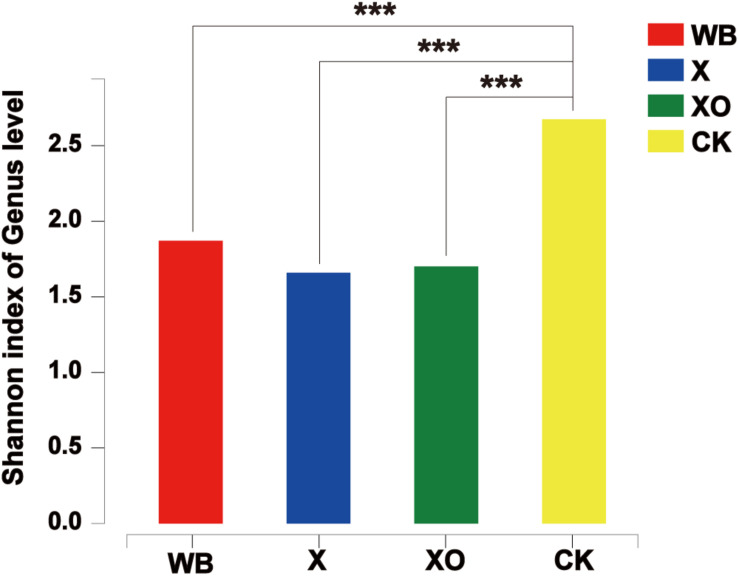
The Shannon diversity index of microbial communities under each treatment at 24 h. Significance was determined between WB and CK, X and CK, and XO and CK using Student’s *t*-test; ***P < 0.001. WB, wheat bran; X, xylan; XO, xylooligosaccharides.

To examine the direct effects of WB, X, and XO on the intestinal microbiota, 16S rRNA sequencing was performed to analyze the microbiota community. Microbial diversity information was obtained through high-throughput sequencing technologies, including DNA fragment extraction, specific primer amplification, and sequencing. A total of 1,824,489 sequences were produced from 40 fecal samples and were classified into 402 OTUs after conducting quality control with 97% similarity (Supplementary Table S2). The alpha diversity of the microbial communities was assessed and calculated (Supplementary Table S3). The ranked abundance curves (Supplementary Figure S3) indicated that the species in the samples were rich and even. The Shannon diversity index of microbial communities showed that the three treatments of *in vitro* batch fermentation lowered the alpha diversity of the human gut microbiota (Figure 3).

**FIGURE 4:**
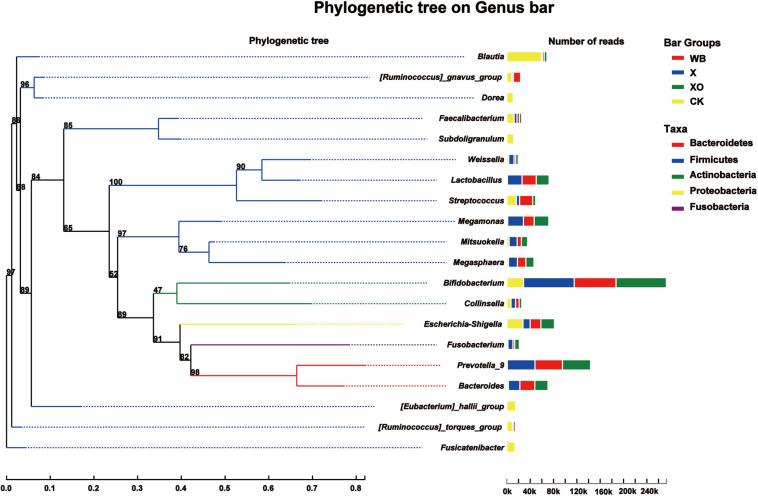
Phylogenetic tree analysis. On the left is the phylogenetic tree. The different colors of the branches of the phylogenetic tree represent different phyla: red (Bacteroidetes), blue (Firmicutes), green (Actinobacteria), yellow (Proteobacteria), and purple (Fusobacteria). The length of the branch is the evolutionary distance between two species, namely, the degree of species difference. The right histogram shows the average number of reads belonging to different genus in each treatment. WB, wheat bran; X, xylan; XO, xylooligosaccharides.

The phylogenetic tree on the genus bar shows the top 20 species in the total level of classification and reveals the phylogenetic order of the classification in the evolution process from the perspective of molecular evolution. At the genus level (Figure 4), the WB, X, and XO treatments increased the number of reads of *Bifidobacterium* (phylum: Actinobacteria), *Prevotella_9* (phylum: Bacteroidetes), *Lactobacillus* (phylum: Firmicutes), *Megamonas* (phylum: Firmicutes), *Bacteroides* (phylum: Bacteroidetes), and *Megasphaera* (phylum: Firmicutes). However, the number of reads of *Blautia*, *Dorea*, *Faecalibacterium*, *Subdoligranulum*, *Escherichia-Shigella*, *[Eubacterium]_hallii_group*, *[Ruminococcus]_torques_group*, and *Fusicatenibacter*, who all belong to the Firmicutes phylum, decreased. The number of reads of *Streptococcus* (phylum: Firmicutes) in the X and XO treatments decreased, but there was no significant change under the WB treatment. In summary, these treatments enriched the Actinobacteria and the Bacteroidetes phyla and worsened the Firmicutes phylum (Supplementary Figure S4).

Supplementary Figure S5 shows the relative abundance of probiotics at the genus level in the WB-, X-, and XO-treated fermentation and the CK. Compared to the CK group, the relative abundance of beneficial bacteria, such as *Bifidobacterium*, *Prevotella_9*, *Lactobacillus*, *Megamonas*, *Bacteroides*, and *Megasphaera*, is significantly increased after *in vitro* fermentation. However, the abundance of beneficial bacteria with WB-treated fermentation is slightly lower than those of the other two treatment groups. This should be related to the polymerization of WB, X, and XO. X and XO are easier to be fermented due to low polymerization, resulting in higher abundances of beneficial bacteria.

**FIGURE 5:**
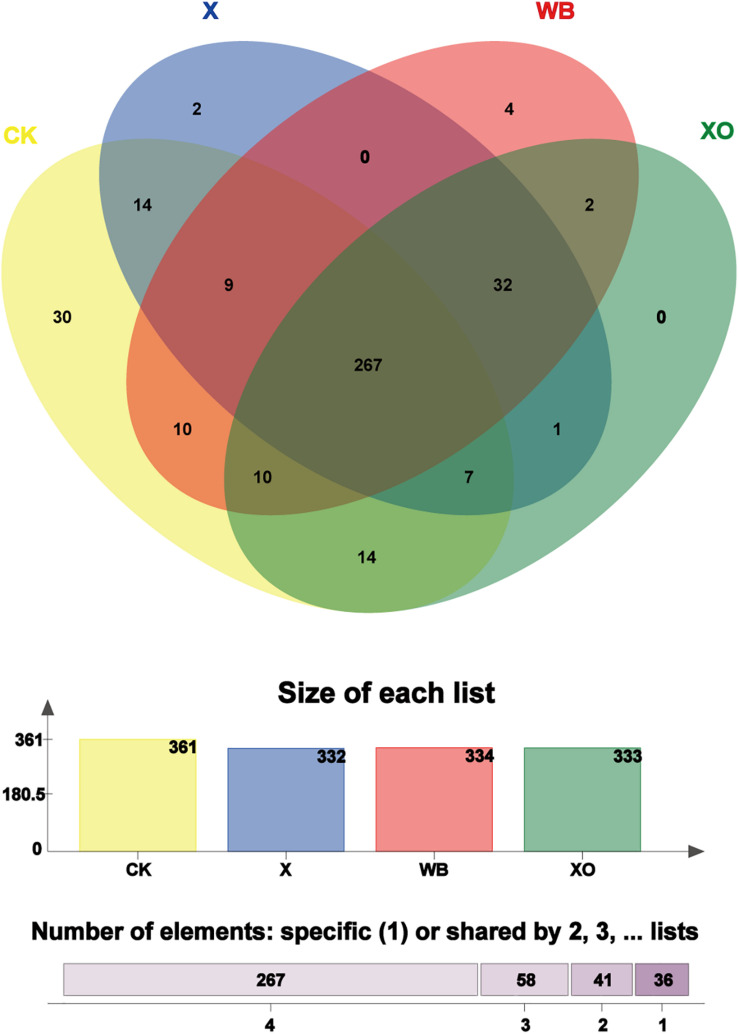
Venn diagram analysis; the total number of core operational taxonomic units (OTUs) shared and unique. “Size of each list” means the number of core OTUs from each treatment. “Number of elements” means the total number of shared core OTUs by two, three, and four groups or the total number of specific core OTUs in each treatment. Different colors represent different treatments, overlapping parts represent species that are common among multiple treatments, parts that do not overlap represent species that are specific to the treatment, and numbers indicate the number of corresponding species. WB, wheat bran; X, xylan; XO, xylooligosaccharides.

To visualize the number of shared and unique OTUs in the three treatments and CK, it is convenient to compare the effects of WB, X, and XO treatments on the *in vitro* fermentation of intestinal microbes; to explore the correlation of substrates with the intestinal microbiota, core OUT analysis was performed (Figure 5). The number of core OTUs in the WB-, X-, and XO-treated fermentation groups and the CK group, which is shown as a bar chart, was 334, 332, 333, and 361, respectively. There were four, two, zero, and 30 core OTUs confined to each of these three treatments and CK, respectively. A vast majority of the core OTUs (267) were shared with all treatments, and 299 core OTUs were shared among WB, X and XO treatments. Interestingly, most XO treatment core OTUs (319/333 OTUs) were shared with the other two treatments’ core OTUs.

Furthermore, the representation of beta diversity of communities presented in the CK and the three treatments fermentation by 3D-PCA (Supplementary Figure S6) shows that these three treatments induced the same variation in the microbiota community composition. This indicates that the components of WB that are mainly digested by intestinal microbes are X and XO.

**FIGURE 6:**
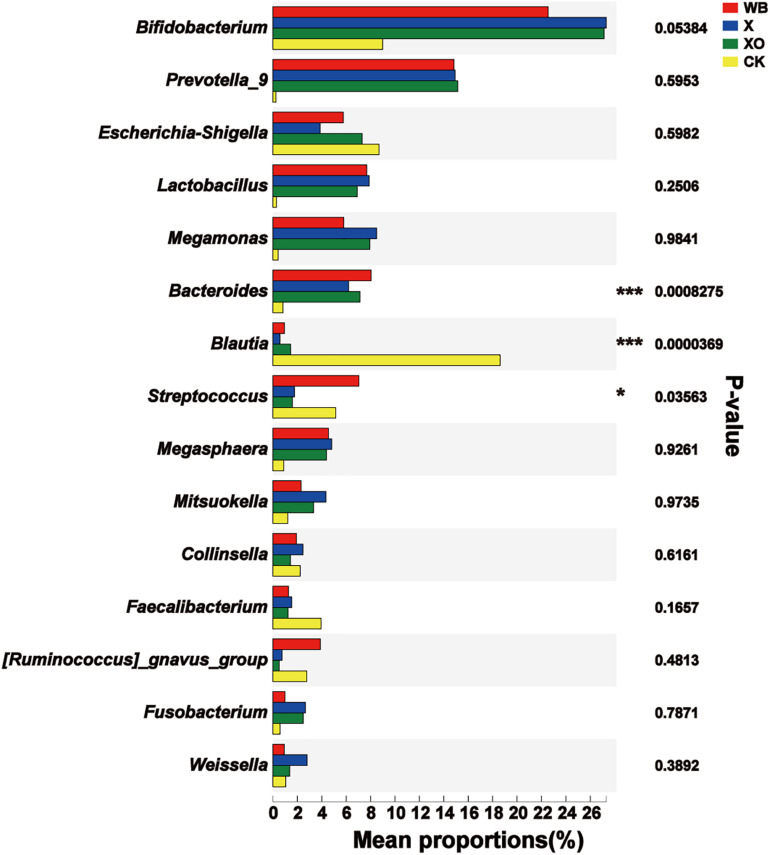
Significant difference test between groups. WB, wheat bran; X, xylan; XO, xylooligosaccharides. Using Kruskal–Wallis *H*-test, the vertical axis represents the species name at the genus level, the column length corresponds to the average relative abundance of the species in each sample group, and the different colors indicate different groupings. The rightmost side is the *P*-value; 0.01 < ^∗^*P*≤0.05, ^∗∗^*P*≤0.01, ^***^P≤0.001.

For the intestinal microbiota fermented with WB, X, or XO, the significant difference in the dominant microbiota was analyzed. Remarkably, there was a significant difference in the Firmicutes and the Bacteroidetes phyla among the four groups (Supplementary Figure S7A). At the genus level (Figure 6), *Bacteroides* was enriched in the three treatment groups (WB, X, and XO), all compared to the control, and *Blautia* was significantly decreased, reflecting common features of dietary fiber diet. However, significant differences between the three treatment groups were in the abundance of *Dorea*, *Bilophila*, and *Sulfurovum*, and all three were the most abundant in the WB treatment group (Supplementary Figure S7B).

In the Circos sample–species relationship diagram (Supplementary Figure S8), the components of the predominant genus microbiota in the different samples were similar, namely, the dominant genus was *Bifidomicrobiota*, as in the three treatment groups of WB, X, and XO, and the relative abundance of *Bifidomicrobiota* increased from 10 to 26, 32, and 32%, respectively. *Prevotella_9* was the second most dominant genus. In the WB, X, and XO treatment groups, the relative abundance of *Prevotella_9* increased from 0.56 to 33, 33, and 34%, respectively. Compared with the CK group, the number of *Lactobacillus* increased from 1.3 to 34, 35, and 30%, respectively. In contrast, the abundance of *Blautia* was significantly reduced from 86 to 4.4, 2.6, and 6.7%, respectively.

**FIGURE 7:**
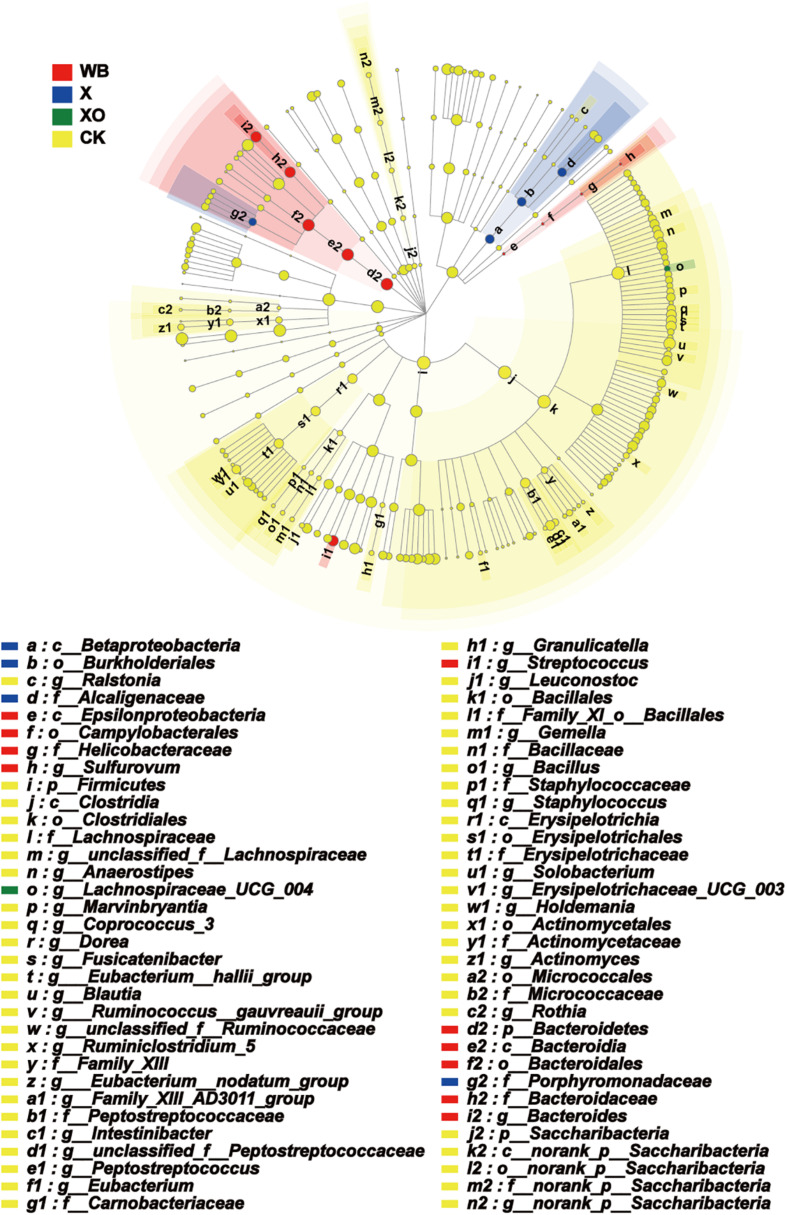
Phylogenetic distribution of the microbiota lineages in the four groups. WB, wheat bran; X, xylan; XO, xylooligosaccharides. The different color nodes represent microbiota communities that are significantly enriched in the corresponding groups and significantly influence the differences between the groups; the pale yellow nodes indicate that the microbial groups either have no significant effect on the different groups or have no significant effect on the differences between groups.

The enriched microbiota in the three treatment groups is shown in cladograms (Figure 7). In the WB treatment, three groups of microbiota were significantly enriched, namely, Bacteroidetes (from phylum to genus, d2–i2), *Streptococcus* (genus, j1), and Epsilonproteomicrobiota (from class to genus, e–h). In the X treatment, two groups of microbiota were significantly enriched, namely, Betaproteomicrobiota, Burkholderiales, Alcaligenaceae (from class to family, a–d), and Prophyromonadaceae (family, g2) were enriched. In the XO treatment, only *Lachnospiraceae_UCG_004* (genus) was detected to be significantly enriched, and no significant enrichment was detected at any other clade. Xylose is the basic unit structure of X and XO, and the difference between X and XO is their molecular weight; Figure 3 also shows that the effect of X and XO on the gut bacteria diversity was similar. WB contains dietary fiber, proteins, and some trace elements, i.e., WB composition is more complicated than X and XO. Figure 7 shows that the number of groups with enriched microbiota in the WB treatment was higher than that in the X and XO treatments; the diversity of gut microbiota in the X and XO treatments was also lower than that in the WB treatment (Figure 3).

Furthermore, Phylogenetic Investigation of Communities by Reconstruction of Unobserved States normalizes the OTU abundance table, obtains the COG family information matching the OTU, and computes the abundance of each COG. The COG functional composition of all samples was similar with the microbiota composition of the CK group (Supplementary Figure S9). Additionally, there was no significant difference in the abundance of COG functions. Above all, the three treatments did not influence the functions of the gut microbiota. Carbohydrate transport and metabolism (G), transcription (K), and amino acid transport, and metabolism (E) were the most abundant functions in all samples.

### FA Content Analysis

FA production following WB-treated fermentation was analyzed, compared with the CK group levels. After removing non-carbohydrate components, such as proteins, in WB, the actual maximum FA conversion rate was 93.5%. Thus, the actual FA production was calculated as the percentage of total carbohydrates in WB. Based on the content of cellulose and hemicellulose in WB, the percentage of contribution of cellulose and hemicellulose to the production of FA was calculated. The statistical results are shown in Table 1.

**TABLE 1:** The concentration of FA production.

No.	WB-CK (μg/mL)	FA/WB (g/Kg)	Actual conversion rate (0.935 g/Kg bran)
1	1.02	10.16%	10.86%
2	0.24	2.41%	2.58%
3	1.73	17.32%	18.53%
4	2.20	21.96%	23.49%
5	2.22	22.22%	23.77%
6	0.46	4.57%	4.88%
7	1.82	18.25%	19.52%
8	0.99	9.92%	10.61%
9	0	0	0
10	0.90	8.95%	9.58%

*WB and CK represent the FA production before and after WB-treated fermentation; WB-CK represents the net FA production after WB-treated fermentation; and FA/WB (g/Kg) represents the percentage of FA produced after fermentation to the total FA content.*

Based on the differences in intestinal microbiota between individuals, it can be seen that samples no. 4 and no. 5 had the highest FA yields of 21.96 and 22.22%, respectively. According to the total conversion rate of FA in the WB treatment, the actual FA conversion rates were 23.49 and 23.77%, respectively. To mine the enzymes that produce FA in the human gut microbiota, we selected the sample with the highest FA yield (5_WB) among the 20 samples and sent it to Majorbio (Shanghai, China) for metagenome sequencing.

### Metagenomic Analysis

The original sequences of 5_WB were subjected to data quality control, and the optimized sequence number was 86423270. The number of sequences of splicing assembly contigs was 133,192. The number of ORFs obtained by gene prediction was 247,049. The number of genes in the non-redundant gene set was 921, and the corresponding abundance information was counted. Through species and function annotations, the annotation information of sample 5_WB was obtained at various taxonomic levels, and an overview of the annotation of carbohydrate-active enzyme genes is provided.

**FIGURE 8:**
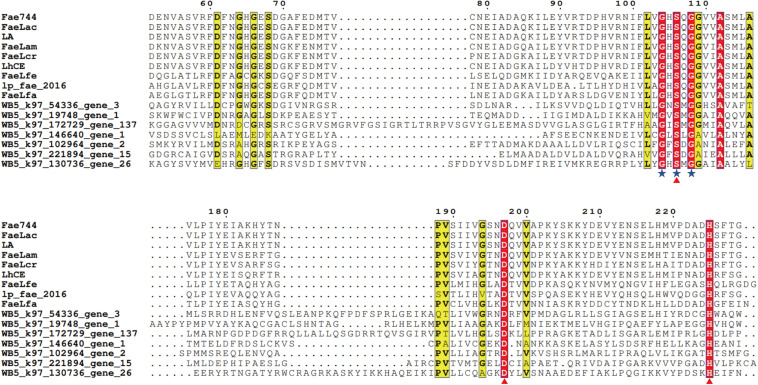
Comparison of the conserved residues, Gly-X-Ser-X-Gly, and the catalytic triplet, Ser-His-Asp. Multiple alignment showing seven novel amino acid sequences with seven known ferulic acid esterase amino acid sequences. The blue asterisk indicates the conserved residues, G-X-S-X-G, and the triangle refers to the catalytic triplet, Ser-His-Asp. The sequences were obtained from Entry ID KX545370 (*Lactobacillus amylovorus CGMCC 11056*), Entry ID MK640209 (*Lactobacillus crispatus S524*), Entry ID KJ596484 (*Lactobacillus helveticus KCCM 11223*), Entry ID KX545371 (*Lactobacillus acidophilus CCTCC AB2010208*), Entry ID YP_004890534 (*Lactobacillus plantarum TMW 1.460*), Entry ID KX545372 (*Lactobacillus farciminis CCTCC AB2016237*), and Entry ID KX545373 (*Lactobacillus fermentum CCTCC AB2010204*) corresponding to Fae 744, FaeLac, LA, FaeLam, FaeLcr, LhCE, FaeLfe, lp_fae_2016, and FaeLfa.

By searching FAE information, 213 genes belonging to CE1 in the CAZy database were obtained. We then created a gene set from the obtained 213 genes and performed species and function annotations. Among these genes, 24 genes had the same functional characteristics of α/β hydrolase as FAEs. According to the source of 24 gene annotated species, five categories were obtained (Bacilli, Lachnospiraceae, Enterobacteriaceae, Faecalibacterium, others). These 24 genes were analyzed for homology with FAEs of the same origin that have been published. According to the two characteristics of FAEs, the conserved residues, Gly-X-Ser-X-Gly, and the catalytic triad, Ser-His-Asp, we used clusterX2 software and ESPript 3.0 website^7^ to perform homologous protein sequence alignment analysis (Robert and Gouet, 2014). The results of the comparison revealed that seven of the 24 genes have two FAE characteristics (Figure 8) and are thus potential FAE genes.

## Discussion

WB is rich in dietary fiber, which can be divided into hexoses and pentoses. Previous research was focused on the *in vitro* effect of different degrees of polymerization on the gut bacteria. That study showed that the degree of hexose polymerization was proportional to the production of butyric acid and the relative abundance of the pathogenic bacteria, *Escherichia–Shigella*. In contrast, the degree of hexose polymerization inversely correlated with the relative abundance of the probiotics, *Bifidobacterium* and *Lactobacillus* (Chen et al., 2020). In this study, the effect of X, XO, and WB on the gut bacteria was researched. Furthermore, the similarities and the differences in the effects of the three treatments were examined in detail, and potential FAE genes were screened.

A previous study indicated that arabinoxylan oligosaccharides may be the key to the beneficial effects of WB on the intestinal microbiota (Suriano et al., 2017). X and XO are the main compounds of the WB dietary fiber, accounting for more than 70%. As prebiotics, the X and XO treatments shared 80% core OTUs with WB treatment. Additionally, compared with the CK group (Figure 6), the relative abundance of probiotics (Burnet and Cowen, 2013; Yoshida et al., 2018; Vuillermin et al., 2020), such as *Bifidobacterium*, *Prevotella*_9, *Lactobacillus*, and *Bacteroides*, was increased by WB, X, and XO treatments. Furthermore, the microbial community composition largely confirmed the bifidogenic effect of the three substrates (D’Hoe et al., 2018), which indicated that WB has a similar prebiotic function to that of X and XO.

Another reason for differences in fermentation characteristics and gut microbial community among WB-, X- and XO-treated fermentation is the composition of WB. Except for polysaccharides of the most important classes, WB also contains proteins and some trace elements (i.e., Broekaert et al., 2011); hence, in addition to the prebiotic effects, there are several other effects on gut bacteria. An analysis of the significant differences among the WB-, X- and XO-treated fermentations revealed that the relative abundance of *Dorea*, *Bilophila*, and *Sulfurovum* in the WB treatment was higher than that in the X and XO treatments. *Dorea* is a hydrogen producer (Zhou et al., 2020), *Bilophila* is a potential pathogen and detrimental bacteria (Toe et al., 2020), and *Sulfurovum* is related to sulfur metabolism (Mori et al., 2018). Furthermore, it has been reported that WB promoted steatosis and adipose tissue inflammation (Suriano et al., 2017). Intriguingly, Epsilonproteomicrobiota, a significantly enriched group in the WB treatment, is widely known for its pathogenic genera *Campylobacter*, *Helicobacter*, and, to a lesser extent, *Arcobacter* (Waite et al., 2017).

In addition to containing X and XO which account for 74% of dietary fiber, WB also contains other dietary fiber components such as β-glucan and galactooligosaccharide. Previous research results showed that β-glucan and galactooligosaccharides in WB could significantly enrich *Bifidobacterium*, *Bacteroides*, and *Megasphaera* population (Chen et al., 2020). Sophie Fehlbaum used the *in vitro* fermentation method to study the effect of different substrate concentrations on the enrichment of *Bifidobacterium* and *Lactobacillus* and found that the optimal substrate concentrations for galactooligosaccharides, XO, and β-glucan were 0.8, 0.8, and 0.2% (w/v), respectively (Fehlbaum et al., 2018). Another reason for higher total gut bacteria diversity in WB as substrate than X and XO should be related to the slower fermentation of WB, stimulating the growth of bacteria continuously.

Moreover, the metabolite production explained the difference in the three treatments. Dietary fiber has a strong influence on the composition of intestinal microbiota and metabolites, mainly the typical SCFAs, acetic acid, propionic acid, and butyric acid (De Filippo et al., 2010; Tremaroli and Backhed, 2012). Acetic acid, the major SCFA, has beneficial effects on glucose tolerance and insulin secretion in high-fat diet-fed rats (Cani et al., 2007). Butyric acid is used preferentially as an energy source by the gut mucosa and has trophic and anti-inflammatory effects on epithelial cells, while propionic acid contributes to gluconeogenesis in the liver (Morrison and Preston, 2016).

WB stimulated propionate and butyrate production (Duncan et al., 2016; De Paepe et al., 2018; Tuncil et al., 2018). Similarly, we can draw the same conclusion from previous studies that the XO treatment increased the yield of butyric acid and acetic acid (Miguez et al., 2018). There was no significant difference in the yields of propionic acid and butyric acid between WB, X, and XO treatments.

Furthermore, the gut microbiota can metabolize certain complex, plant-derived carbohydrates that are common in the (adult) human diet. While few studies have focused on FAEs, Kelly et al. (2018) have discovered a new hydroxycinnamic acid esterase that may be beneficial for the gut environment *via Bifidobacterium longum* subsp. *longum*. Using metagenomic sequencing, we found seven novel potential FAE genes. Our results provide a basis for increasing the extraction rate of FAs from WB and other cereal brans to improve the nutritional value of WB. In this study, seven potential FAE genes in the human gut microbiota were screened.

Although humans cannot secret endogenous enzymes to degrade dietary fibers in the foregut, the structure and properties of dietary fibers are affected due to the gut environment, such as gastric acid. Song et al. (2019) illustrate that dietary fiber was resistant to artificial gastric acid (pH 2) with no more than 4% (w/w) hydrolysis in 6 h, and our purpose is to study the key to probiotics of WB on human gut microbiota so that the method used in this study was *in vitro* fermentation. The related literature has reported that the *in vitro* static fermentation model is limited by nutrients and microbiota metabolites and thus cannot reflect the entire colonic microbiota (Wang et al., 2019). However, as we expected, the low amounts of dietary fiber in human intervention studies are consistent with the *in vitro* results (Sasaki et al., 2018). Additionally, we did not treat the fibrous substrate before the *in vitro* fermentation because this study aimed to investigate the natural effect of the substrate on human gut microbiota *in vitro*. Moreover, we tested the data at a single time point after fermentation as there was no significant difference between 24 and 48 h in the pre-test. Consequently, we selected 24 h, which is the time required to digest food in the body, to capture a picture of the influence of substrates on gut microbiota.

## Conclusion

*In vitro* fermentation with X, XO, and WB treatments demonstrated that WB has prebiotic functions and effects on the gut bacteria, such as Epsilonproteomicrobiota. This study also screened seven FAE genes, which provided a theoretical basis for exploring the mechanism of action of FAEs in the gut bacteria.

## Data Availability Statement

The raw reads used in this study have been deposited into the NCBI Sequence Read Archive (SRA) (Accession number: PRJNA577201).

## Author Contributions

MC, SL, BW, and FX designed the study. MC and SL conducted *in vitro* fermentation and culturing, measured pH and air pressure, quantified SCFA concentrations, and performed DNA extraction. MC conducted physicochemical property analysis, bioinformatics, and all statistical analysis. BW, MC, and FX interpreted the results. MC drafted the manuscript with contributions of SL and FX. BW and KI contributed to writing—reviewing and editing. BW conceptualized this study. Visualization, supervision, project administration, and funding acquisition were the responsibility of FX. BW, FX, MC, SL, KI, LS, YW, and TG read and approved the final manuscript.

## Conflict of Interest

The authors declare that the research was conducted in the absence of any commercial or financial relationships that could be construed as a potential conflict of interest.

## Footnotes

1 https://github.com/jstjohn/SeqPrep

2 https://github.com/najoshi/sickle

3 http://bio-bwa.sourceforge.net

4 http://soap.genomics.org.cn/

5 http://hmmer.janelia.org/search/hmmscan

6 http://www.cazy.org/

7 http://espript.ibcp.fr/ESPript/ESPript/
