# Supplementary material for: The Effect of Xylooligosaccharide, Xylan, and Whole Wheat Bran on the Human Gut Bacteria
Source: Front Microbiol. 2020 Dec 3;11:568457. doi: 10.3389/fmicb.2020.568457 (PMC7794011; doi:10.3389/fmicb.2020.568457)

The Effect of Xylooligosaccharide, Xylan and the Whole Wheat Bran on the Human Gut Bacteria

**Supplementary material**

Table S1. Polymeric carbohydrates and hemicellulose content in wheat bran.

|  | **Polymeric carbohydrates** | **Hemicellulose** |
| --- | --- | --- |
| Parallel 1 | 17.498% | 10.439% |
| Parallel 2 | 17.577% | 10.652% |
| Parallel 3 | 18.571% | 10.174% |
| Average | 17.88% | 10.42% |
| STDEVP | 0.49 | 0.20 |

Table S2. Taxon statistics

| **Amplified Region** | **Samples** | **Sequences** | **Bases(bp)** | **Average length** |
| --- | --- | --- | --- | --- |
| 338F_806R | 40 | 1824489 | 800658283 | 438.84 |

Table S3. Alpha-diversity of gut microbiota in fecal samples

| **Sample** | **Shannon** | **Simpson** | **Chao** | **Coverage** |
| --- | --- | --- | --- | --- |
| 1_XO | 1.093 | 0.404 | 6.000 | 1.000 |
| 2_XO | 1.513 | 0.233 | 6.000 | 1.000 |
| 3_XO | 0.951 | 0.499 | 6.000 | 1.000 |
| 4_XO | 1.257 | 0.309 | 6.000 | 1.000 |
| 5_XO | 0.947 | 0.437 | 5.000 | 1.000 |
| 6_XO | 0.801 | 0.578 | 5.000 | 1.000 |
| 7_XO | 1.020 | 0.429 | 7.000 | 1.000 |
| 8_XO | 1.043 | 0.423 | 5.000 | 1.000 |
| 9_XO | 0.845 | 0.546 | 8.000 | 1.000 |
| 10_XO | 1.051 | 0.397 | 6.000 | 1.000 |
| 1_X | 0.921 | 0.515 | 5.000 | 1.000 |
| 2_X | 1.462 | 0.251 | 5.000 | 1.000 |
| 3_X | 1.208 | 0.326 | 4.000 | 1.000 |
| 4_X | 1.064 | 0.398 | 9.000 | 1.000 |
| 5_X | 0.755 | 0.532 | 6.000 | 1.000 |
| 6_X | 0.788 | 0.593 | 5.000 | 1.000 |
| 7_X | 0.678 | 0.653 | 6.000 | 1.000 |
| 8_X | 1.053 | 0.405 | 5.000 | 1.000 |
| 9_X | 0.782 | 0.555 | 8.000 | 1.000 |
| 10_X | 1.051 | 0.423 | 7.000 | 1.000 |
| 1_WB | 1.262 | 0.307 | 5.000 | 1.000 |
| 2_WB | 1.214 | 0.362 | 7.000 | 1.000 |
| 3_WB | 1.010 | 0.435 | 6.000 | 1.000 |
| 4_WB | 1.207 | 0.338 | 7.000 | 1.000 |
| 5_WB | 1.033 | 0.429 | 5.000 | 1.000 |
| 6_WB | 0.618 | 0.723 | 5.000 | 1.000 |
| 7_WB | 1.053 | 0.434 | 5.000 | 1.000 |
| 8_WB | 1.163 | 0.353 | 6.000 | 1.000 |
| 9_WB | 1.131 | 0.343 | 10.000 | 1.000 |
| 10_WB | 0.993 | 0.478 | 7.500 | 1.000 |
| 1_CK | 0.628 | 0.712 | 11.000 | 1.000 |
| 2_CK | 0.902 | 0.525 | 8.000 | 1.000 |
| 3_CK | 0.461 | 0.774 | 5.000 | 1.000 |
| 4_CK | 0.479 | 0.771 | 8.000 | 1.000 |
| 5_CK | 0.958 | 0.439 | 6.000 | 1.000 |
| 6_CK | 0.480 | 0.770 | 7.000 | 1.000 |
| 7_CK | 0.833 | 0.509 | 6.000 | 1.000 |
| 8_CK | 0.912 | 0.440 | 6.000 | 1.000 |
| 9_CK | 0.688 | 0.621 | 8.000 | 1.000 |
| 10_CK | 0.532 | 0.743 | 8.000 | 1.000 |

**Figure S1.** Barometric pressure of in-vitro fermentation after 24 h. The significant difference in barometric pressure was calculated by Kruskal–Wallis test; *** P < 0.001 compared with the control.


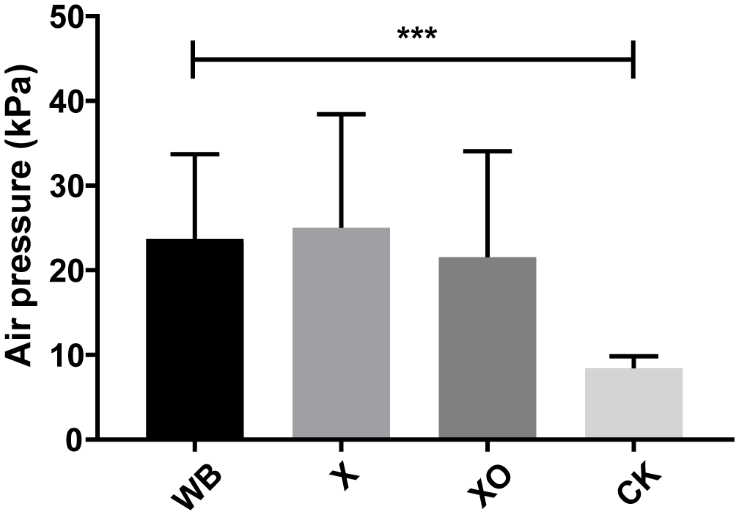


**Figure S2.** The concentration (μmol/g) of SCFAs in in vitro fermentation. The production of acetic acid (A), propionic acid (B), butyric acid (C), and total SCFAs production (D) was measured. The significant difference for each acid was calculated by Kruskal–Wallis test; ** P < 0.01, **** P < 0.0001 and “ns” (no significant difference) compared with the CK group.

| A  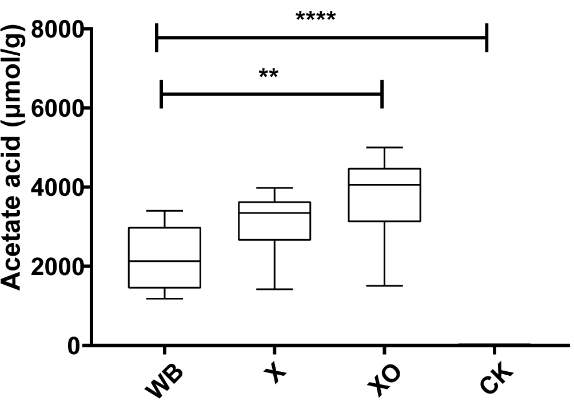 | B  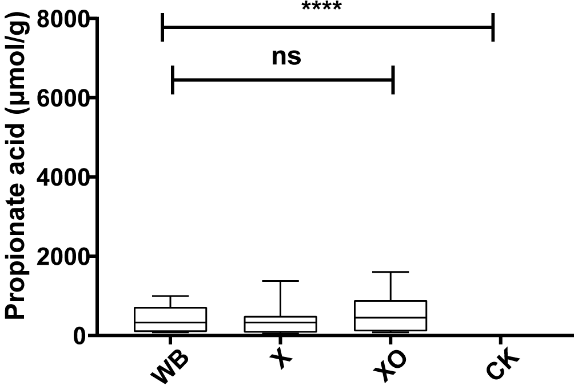 |
| --- | --- |
| C  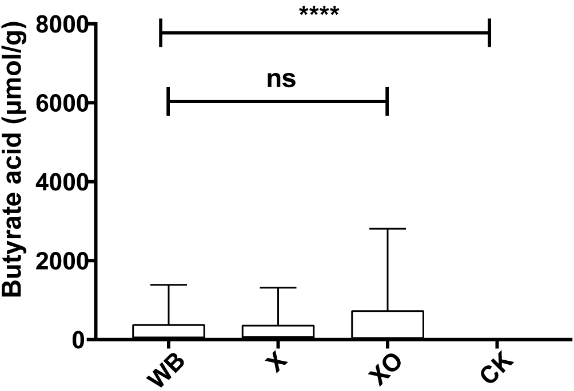 | D  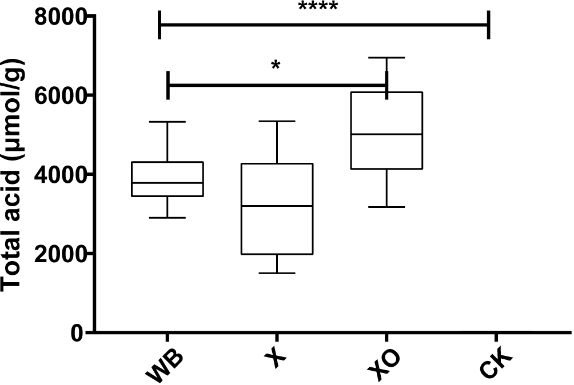 |

**Figure S3**. Rank-abundance curves derived from the OTU level.


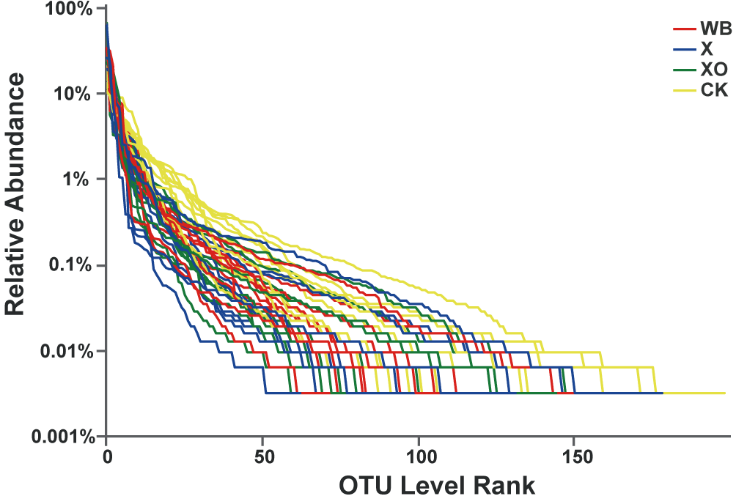


**Figure S4**. Phylogenetic tree at the phylum level. On the left is the phylogenetic tree. Each branch in the phylogenetic tree represents a species. The length of the branch is the evolutionary distance between the two species, namely the degree of species difference. The right histogram shows the number of reads belonging to different species in each treatment.


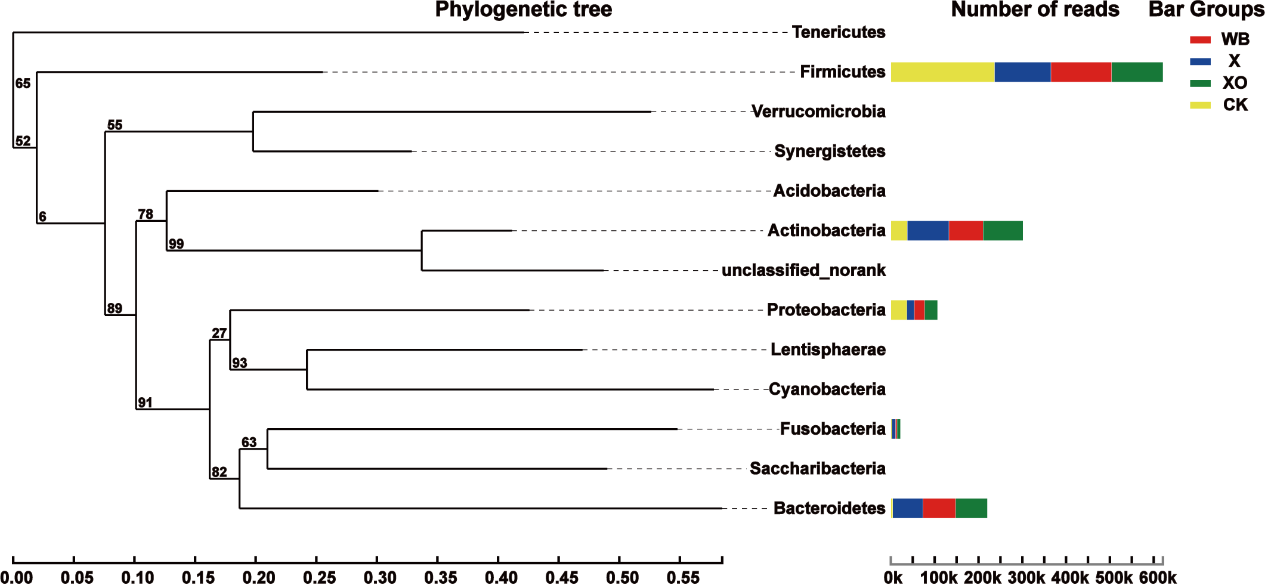


**Figure S5.** Relative abundance of probiotics at the genus level in the wheat bran (WB), xylan (X), xylooligosaccharides (XO) and the control group (CK).


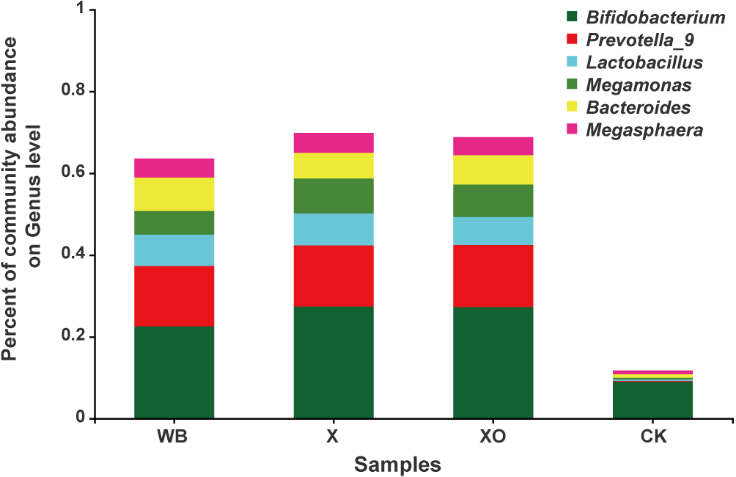


**Figure S6**. Principal Component Analysis (PCA) based on euclidean among the samples of wheat bran (WB), xylan (X), xylooligosaccharides (XO) and the control group (CK).


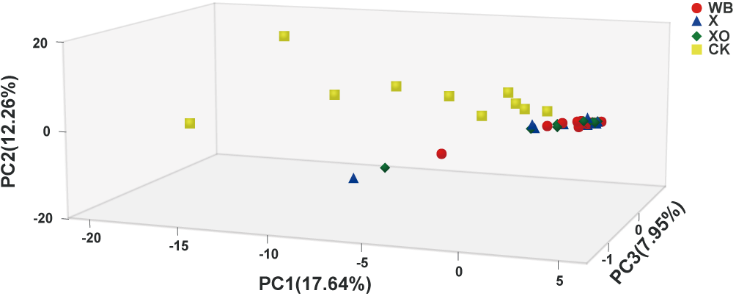


**Figure S7**. Significant difference test comparing the four groups at the phylum level (a) and comparing the three treatments at the genus level (b). Using Kruskal–Wallis H test, the vertical axis represents the species name at the genus level, and the column length corresponding to the average relative abundance of the species in each sample group. The different colors indicate different groupings. The rightmost side is the P value, * 0.01 < P ≤ 0.05, ** 0.001 < P ≤ 0.01, *** P ≤ 0.001.

| a.  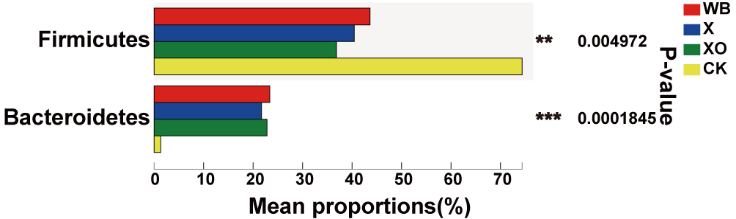 | b.  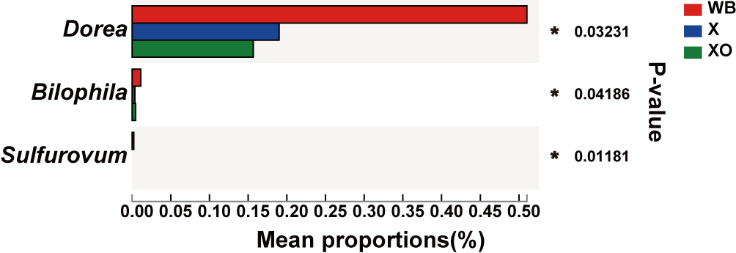 |
| --- | --- |

**Figure S8.** Relationship between samples and genus-species abundance. The left half-circle indicates the species composition in the samples; the color of the outer ribbon on the left represents the group from which sample; the color of the inner layer is the species, and the length represents the relative abundance of the species in the corresponding sample. The large semicircle indicates the distribution ratio of species in different samples at the genus level. The outer ribbon represents the genus species, the inner color of the ribbon represents different groups, and the length represents the proportion of the sample in each species.


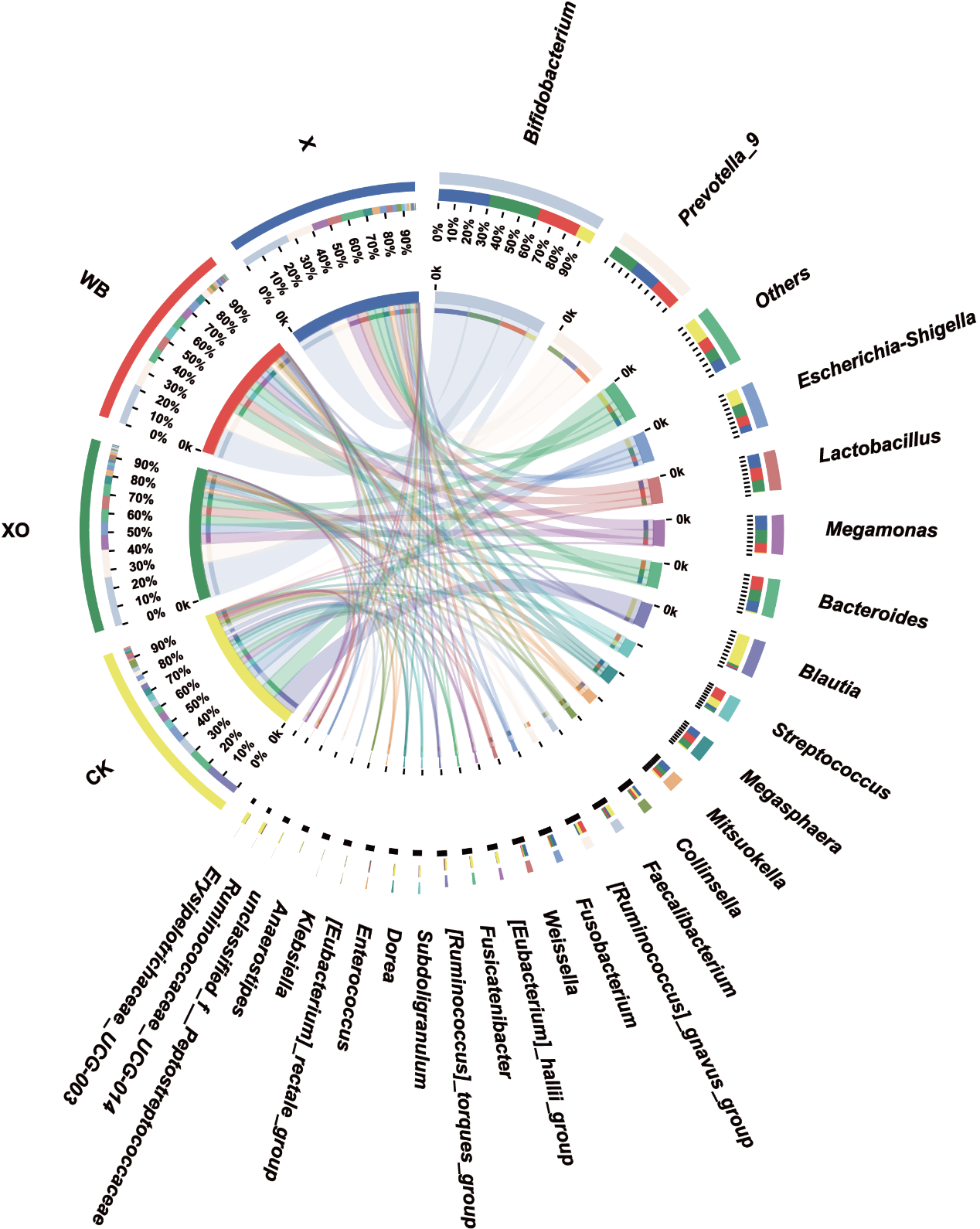


**Figure S9.** 16S function abundance analysis. WB, wheat bran; X, xylan; XO, xylooligosaccharides. Relative abundance represents the ratio of each function abundance in the sample to the full functional abundance of the sample.


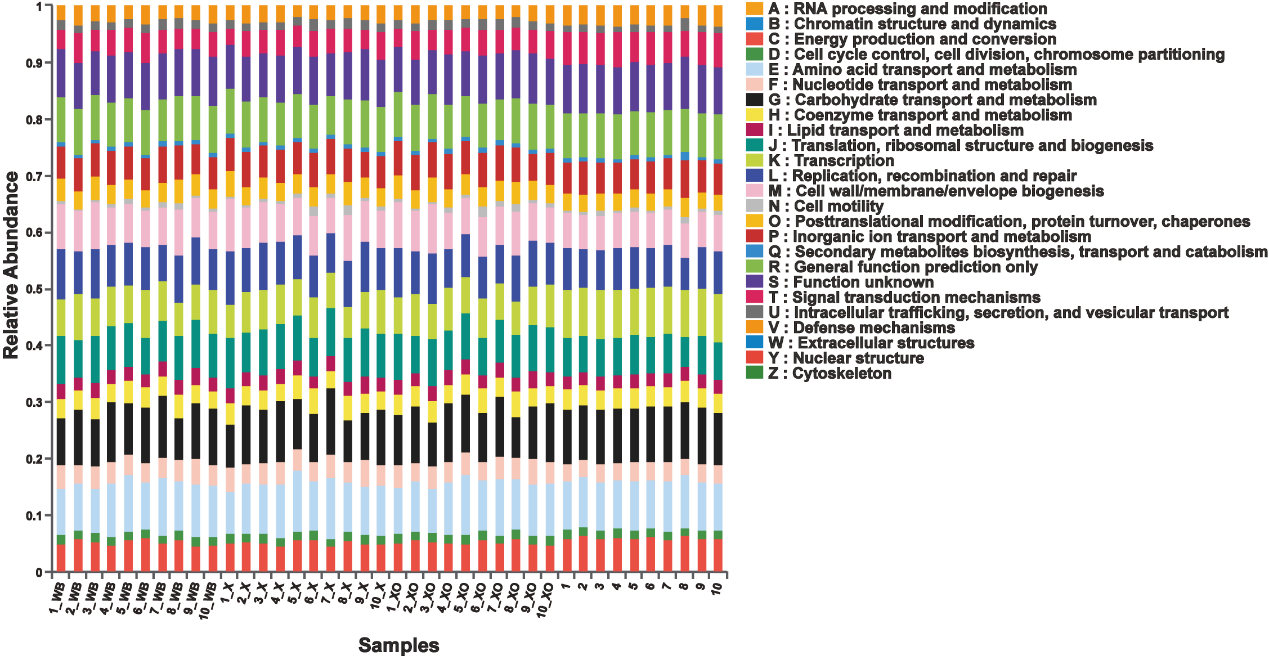

Supplement: Supplementary file 1 [file Data_Sheet_1.docx]
